# Initiation time of double-filtration plasma apheresis affects the risk of persistent organ failure in hypertriglyceridaemia-induced acute pancreatitis: a retrospective study

**DOI:** 10.1038/s41598-023-40287-2

**Published:** 2023-08-10

**Authors:** Wei Su, Yuzhen Gao, Xiaoling Wang, Donghai Wang, Binbin Feng, Yinshan Wu, Huijun Zheng, Feng Guo

**Affiliations:** 1 Department of Critical Care Medicine, Sir Run Run Shaw Hospital, Zhejiang University School of Medicine, Hangzhou, 310016 Zhejiang China; 2 Department of Clinical Laboratory, Sir Run Run Shaw Hospital, Zhejiang University School of Medicine, Hangzhou, 310016 Zhejiang China

**Keywords:** Dyslipidaemias, Pancreatitis, Acute pancreatitis

## Abstract

The effect of double filtration plasma apheresis (DFPP) on improving the outcomes of patients with hypertriglyceridaemia-induced acute pancreatitis (HTG-AP) remains unclear. The aim of this study was to evaluate the relationship between the initiation time of DFPP and the risk of persistent organ failure (POF) in an HTG-AP cohort in China. We retrospectively evaluated data from HTG-AP patients treated with DFPP 48 h after diagnosis between January 2017 and January 2022. Comparisons across tertiles of the interval from diagnosis to completion of one DFPP session (DTD) were analysed. Logistic regression models and restricted cubic splines (RCS) were used to determine the correlation between the DTD time and risk of POF. Of the 89 patients enrolled, 46 patients (51.69%) suffered POF in the first week of HTG-AP. DFPP was initiated at a median of 17 h after the diagnosis was confirmed. The patients in the highest tertile of DTD time had a significantly increased prevalence of POF. After multivariate adjustment, the logistic regression models found a significant decrease in the odds ratios (OR) of POF from the highest to the lowest DTD tertile (P for trend = 0.006). Moreover, the RCS curves showed a nonlinear relationship in the adjusted OR of POF and DTD time, which remained relatively low and flat during the early DTD time but increased sharply afterwards. Early initiation of DFPP treatment correlates with a reduced risk of POF in HTG-AP patients.

## Introduction

Hypertriglyceridaemia (HTG) is one of the main causes of acute pancreatitis (AP) and has recently become the second leading cause of AP in China^1–3^. Some reports have found that hypertriglyceridaemia-induced acute pancreatitis (HTG-AP) is associated with a high rate of complications, such as persistent organ failure (POF), compared to other aetiologies of AP^4,5^. AP patients with POF, defined as severe acute pancreatitis (SAP), still have higher mortality, ICU/hospitalization needs and costs^6–8^. Although the pathophysiology of HTG-AP remains unclear, the prominent theory suggests that excessively elevated serum triglyceride (TG) leading to the accumulation of free fatty acids (FFAs) may cause injury to the pancreas, trigger an inflammatory cascade and result in POF during the first week of disease^4,5,8–11^. Prompt reduction of serum TG is essential in the HTG-AP setting, and it is generally proposed that immediate reduction of TG levels with a target below 5.65 mmol/L (500 mg/dL) is proportionally associated with reduction of POF in HTG-AP^4,11–14^.

Intravenous insulin with or without heparin, and therapeutic plasmapheresis (TPE) are available regimens to reduce serum TG in HTG-AP patients, but there is no consensus on first-line therapy^4,10,12,13^. Intravenous insulin is a non-invasive treatment by increasing activation of lipoprotein lipase (LPL) to accelerate chylomicron breakdown, and would be recommended in patients with concomitant hyperglycemia, especially with metabolic decompensation such as ketoacidosis^12,13^. However, in non-diabetics, the use of insulin, with glucose, has not been proven to be efficacious^12^. TPE is a rapid TG-clearance treatment over other therapies that can directly remove lipids from the circulation and may thus theoretically eliminate the causal factor in HTG-induced pancreatitis^12–15^. It has always been recommended in patients with very high TG levels (> 2000 mg/dL) or in the emergency setting, such as severe HTG-AP complicated with worsening organ dysfunction/multiorgan failure, worsening systemic inflammation, or lactic acidosis^14–19^. Double filtration plasmapheresis (DFPP) is a selective method of TPE^20^. Because DFPP has the advantage of reducing the need for fresh frozen plasma (FFP) as a replacement fluid and significantly reducing the risk of allergic reactions and viral infections secondary to FFP, it has been increasingly used to treat HTG-AP in recent years^20–27^. However, there is little evidence to conclude that DFPP has an effect on improving outcomes of patients with HTG-AP, particularly in terms of reducing organ injury^22–25^. It has been reported that rapid TG clearance by DFPP in the clinical setting may not translate into a theoretical effect^26,27^. As mentioned in the relevant literature, the timing of apheresis treatment may be critical to its effect on the outcomes in the early stage of HTG-AP^4,15,28,29^. Despite this, there is no research on the relationship between the initiation time of DFPP and outcomes in HTG-AP.

In this retrospective study, we reviewed the records of patients who received DFPP treatment within 48 h after the diagnosis of HTG-AP to investigate the effect of the initiation time of DFPP on the development of POF and to determine whether early DFPP treatment has a definite clinical effect on reducing the risk of POF during the early stage of HTG-AP. In addition, we divided the patients into four subgroups based on TG level at diagnosis (pre-TG), sex, body mass index (BMI) and diabetic ketoacidosis (DKA) to explore whether the above relationships could be maintained between the four subgroups.

## Methods

### Study design and patients

The study protocol was reviewed and approved by the Ethics Committee, Sir Run Run Shaw Hospital, Zhejiang University School of Medicine (protocol ID 20220336). Due to the retrospective and observational nature of the study, informed consent was waived as part of the approval by the same ethic committee. All methods were performed in accordance with the relevant guidelines and national regulations. We retrospectively reviewed the electronic medical records of patients who were diagnosed with HTG-AP during hospitalization at Sir Run Run Shaw Hospital from January 2017 to January 2022. We identified potential patients using discharge diagnoses with acute pancreatitis (ICD-10 code for acute pancreatitis [K85]). According to the 2012 Atlanta criteria, the diagnosis of acute pancreatitis requires 2 of the following 3 criteria: (1) upper abdominal pain of acute onset; (2) serum amylase or lipase activity greater than 3 times normal; and (3) findings on cross-sectional abdominal imaging consistent with acute pancreatitis^6^. AP patients who had an admission serum TG level of 11.3 mmol/L (1000 mg/dL) or more on admission were included in the study. The exclusion criteria included patients who had completed one session of DFPP treatment longer than 48 h of the diagnosis; patients who were transferred from another hospital with missed data for analysis; patients less than 18 years old; patients who were pregnancy or who had chronic pancreatitis or other advanced comorbidities, such as severe infectious or immunosuppressive conditions, congestive heart failure 3–4 stage or unstable coronary heart disease, end stage lung diseases, chronic kidney disease stage 4–5, liver cirrhosis with modified Child‒Pugh grade 2–3, active malignancy, or multiple organ dysfunction due to other diseases.

All patients followed our clinical protocols for HTG-AP. The initial treatment plan included fasting, aggressive intravenous hydration, and analgesics. No other TG- clearance treatments were used before DFPP treatment. Low-molecular-weight heparin was only used for thrombosis prophylaxis. Insulin was administered when the glucose level was > 150 mg/dL. DFPP was conducted via the femoral double lumen by a Plasauto EZ machine (Asahi-Kasei, Tokyo, Japan) using a blood cell separator column (Plasmaflo OP-08 W, Asahi Kasei Medical Co, Japan) and a plasma component separator (Cascadeflo EC-40 W, Asahi Kasei Medical Co, Japan). Heparin was used for the anticoagulation of the system. DFPP was available around the clock at our centre. The dosage and frequency of DFPP sessions were decided by the clinicians in each case.

### Data collection

Electronic medical records were reviewed for information on demographics, serial serum TG levels, in-hospital mortality, hospital stay, hospital charges and complications as well as organ support therapies during hospitalization. We collected the Sequential Organ Failure Assessment (SOFA) score acute physiology and chronic health evaluation II (APACHE II) score on admission and the maximum score during the first 48 h after admission. The DFPP procedures were reviewed, and their parameters were recorded as well as any eventual complications during DFPP treatment. At the time of data collection, the collectors were blinded to the outcomes being investigated. Electronic medical records and paper charts were reviewed by two independent physicians to calculate scores, and inconsistent scores had to be recalculated until the same score was reached.

### Definitions

In accordance with the revised Atlanta definition criteria 2012^6^, we assessed 3 organ systems to define organ failure (OF): respiratory, renal, and cardiovascular, which was defined by (1) PaO_2_/FiO_2_ less than 300 mmHg; (2) serum creatinine greater than 170 μmol/L; and (3) systolic blood pressure less than 90 mmHg unresponsive to fluid resuscitation. Persistent organ failure (POF) was defined as OF persisting for more than 48 h. Diagnosis time was defined as the time of the clinical results at first presentation to diagnose HTG-AP, such as serum amylase or lipase and serum TG level. Contrast-enhanced computed tomography was generally performed approximately 3–7 days after AP onset to assess pancreatic necrosis (PNec), defined as pancreatic parenchymal and/or peripancreatic necrosis. Infected pancreatic necrosis (IPN) was confirmed by a positive bacterial or fungal culture or Gram stain of pancreatic or extrapancreatic necrosis obtained by fine-needle aspiration or from the first drainage procedure or the first necrosectomy.

In our hospital, patients who need treatment with DFPP are routinely admitted to the intensive care unit (ICU). Intensive care unit need was thus defined as the requirement for ICU support, including ventilation, CRRT or/and vasopressor. Ventilation need was defined as the requirement for mechanical ventilation or noninvasive ventilation, such as nasal bilevel-CPAP and high-flow nasal cannula. Continuous renal replacement therapy (CRRT) need was defined as the requirement for continuous renal replacement therapy. Vasopressor need was defined as the requirement for vasopressor.

### Data statistics

Continuous variables are presented as medians with interquartile ranges (IQRs). Categorical variables were summarized using frequencies and percentages. The difference between the POF and non-POF groups for baseline variables was determined by univariate analysis. The comparison across the tertiles of the interval from confirmed diagnosis to complete one DFPP session (DTD) was performed using univariate analysis. The univariate analysis included Mann‒Whitney U tests for continuous variables, χ^2^ tests for categorical variables and Bonferroni correction for multiple comparisons.

Logistic regression models were used to test odds ratios (ORs) and confidence intervals (CIs) of POF and its pattern for each DTD time tertile compared with the highest tertile, with gradual adjustment for the other screened factors. The screened factors were selected by an entry-level significance of a P value less than 0.05 in the univariate analysis. Restricted cubic spline (RCS) based on an adjusted multivariate logistic regression model with 3 knots was used to detect the possible nonlinear dependency of the relationship between the risk of POF and DTD time. The relationship of POF and DTD time in different subgroups classified by sex, BMI, pre-TG and DKA as two groups was explored by the final multivariate logistic regression models. In addition, the interaction between the DTD time and the above subgroup variables was determined. A 2-tailed P value of less than 0.05 was considered statistically significant.

All statistical analyses were performed with IBM SPSS Statistics for Windows, Version 26.0 (IBM Corp, Armonk, NY, USA), GraphPad Prism for Windows, Version 8.0 (GraphPad Software, San Diego, CA, USA) and R software, Version 3.6.2 (www.r-project.org).

### Ethics approval and consent to participate

The study was approved by the Ethics Committee of Sir Run Run Shaw Hospital, Zhejiang University School of Medicine (protocol ID 20220336). Because of the retrospective nature of this study, informed consent was waived.

## Results

### Characteristics of the study population

**Figure 1:**
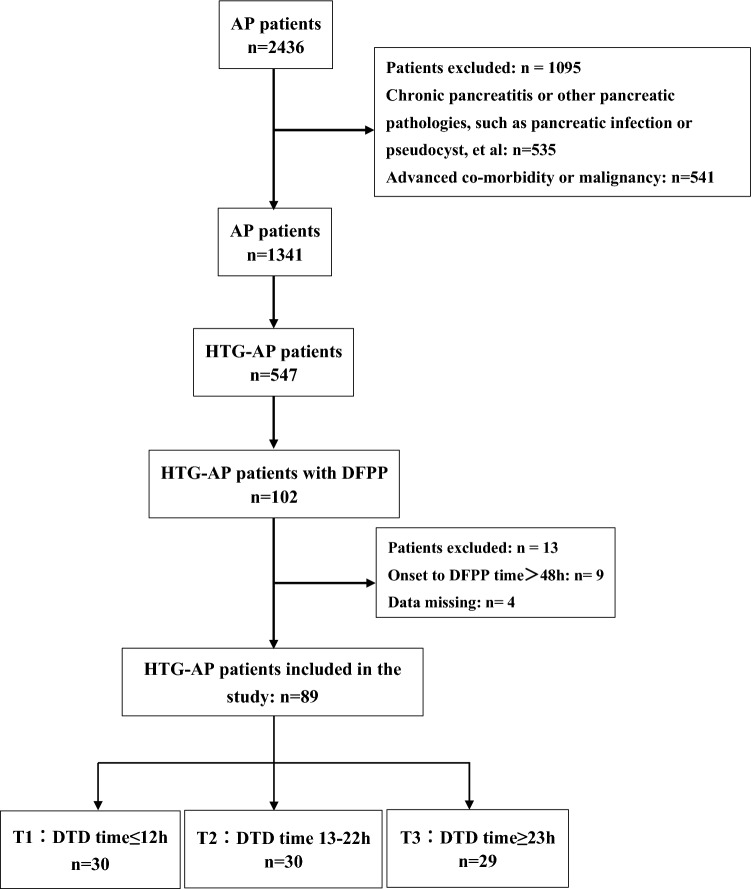
Flowchart of the study. The T1, T2 and T3 groups were classified by tertile interval from diagnosis to completion of one DFPP session. *AP* acute pancreatitis, *HTG-AP* hypertriglyceridaemia-induced acute pancreatitis, *DTD* from diagnosis to completion of one DFPP session.

A total of 89 HTG-AP patients who had completed at least one DFPP session within 48 h of confirmed diagnosis were enrolled. The patient selection process is shown in Fig. 1.

The basic characteristics of the HTG-AP patients sorted by POF and non-POF are shown in Table 1. Of the 89 patients, 23 (25.4%) presented with OF on admission, with no difference between the two groups. The median time interval from diagnosis to completion of one DFPP session (DTD) was 17 h (IQR 11–25 h), and the patients with POF had longer DTD times than the patients without POF (P = 0.002). The POF and non-POF patients also differed significantly in some baseline variables, such as the median Apache II scores on admission and the proportion of hospital transfer (P = 0.002 and P = 0.023). Additional demographic and clinical characteristics between the two groups were not statistically significant.

**Table 1:** Baseline characteristics of the patients with and without POF.

	All patients (n = 89)	Non-POF (n = 43)	POF (n = 46)	P value
Age, median (IQR), years	38 (33–46)	37 (33–47)	38 (31–43)	0.442
Gender, male, n (%)	62 (69.7)	26 (60.5)	36 (78.3)	0.068
BMI, median (IQR), kg/m^2^	27.1 (23.55–29.49)	26 (23.3–29.4)	28.3 (25.8–30.4)	0.053
Diabetes mellitus, n (%)	22 (24.7)	8 (18.6)	14 (30.4)	0.196
Hyperlipidemia, n (%)	15 (16.85)	5 (11.6)	10 (21.7)	0.203
Alcohol abuse, n (%)	11 (12.36)	4 (9.3)	7 (15.2)	0.397
Previous pancreatitis, n (%)	47 (52.81)	21 (48.8)	26 (56.5)	0.468
Hospital transfer, n (%)	40 (44.94)	14 (32.6)	26 (56.5)	**0.023**
Onset to diagnosis, median (IQR), h	12 (8–20.5)	12 (8–21)	12 (6–20)	0.757
Onset to DFPP, median (IQR), h	29 (19–36.5)	28 (19–34)	29 (19–43)	0.274
DTD time, median (IQR), h	17 (11–25)	13 (10–20)	23 (12–28)	**0.002**
TG_0_, median (IQR), mmol/L	58.3 (39.69–80.05)	58.92 (42–81.18)	56.28 (37.11–78.46)	0.197
TG_0_ > 56.5 mmol/L, n (%)	46 (51.69)	23 (53.5)	23 (50)	0.742
OF on admission, n (%)	23 (25.4)	10 (23.3)	13 (28.3)	0.59
SOFA_0_, median (IQR)	1 (0–2)	1 (0–2)	1 (1–2)	0.08
Apache II_0_, median (IQR)	6 (5–9)	6 (4–8)	7 (6–10)	**0.002**

Significant values are in bold.

*APACHE II* acute physiology and chronic health evaluation II score, *APACHE II*_*0*_ APACHE II score on admission, *BMI* body mass index, *DFPP* double filtration plasmapheresis, *DTD* from diagnosis to completion of one DFPP session, *IQR* interquartile range, *OF* organ failure, *POF* persistent organ failure, *SOFA* sequential organ failure assessment, *SOFA*_*0*_ SOFA score on admission, *TG* triglycerides, *TG*_*0*_ the level of triglycerides at diagnosis.

The baseline characteristics of the HTG-AP patients sorted by tertiles of DTD time are shown in Table 2. There were significant differences in the proportion of alcohol abuse and hospital transfer (P = 0.016 and P = 0), the median time from onset to diagnosis (P = 0.001) and SOFA scores on admission (P = 0.014) among the three groups.

**Table 2:** Baseline characteristics of patients according to DTD time tertiles.

	All patients (n = 89)	T1 (n = 30) ≤ 12 h	T2 (n = 30) 13–22 h	T3 (n = 29) ≥ 23 h	P value
Age, median (IQR), years	21 (12–30)	14 (9–22)	18 (12–24)	32 (24–37)	0.576
Gender, male, n (%)	62 (69.7)	18 (60)	21 (70)	23 (79.3)	0.272
BMI, median (IQR), kg/m^2^	27.1 (23.6–29.5)	28.2 (23.3–30)	26.3 (24.2–29)	27.5 (25.1–29.7)	0.546
Obesity, n (%)	41 (46.1)	16 (53.3)	11 (36.7)	14 (48.3)	0.647
Diabetes mellitus, n (%)	22 (24.7)	7 (23.3)	8 (26.7)	7 (24.1)	0.952
DKA, n (%)	56 (62.9)	17 (56.7)	16 (53.3)	23 (79.3)	0.081
Hyperlipidemia, n (%)	15 (16.9)	6 (20)	4 (13.3)	5 (17.2)	0.786
Alcohol abuse, n (%)	11 (12.4)	1 (3.3)	2 (6.7)	8 (27.6)	**0.016**
Previous pancreatitis, n (%)	47 (52.8)	17 (56.7)	19 (63.3)	11 (37.9)	0.129
Hospital Transfer, n (%)	40 (44.9)	3 (10)	16 (53.3)	21 (72.4)	**< 0.001**
Onset to diagnosis, median (IQR), h	12 (8–20.5)	9 (3–16)	11 (8–12)	12 (12–24)	**0.001**
OF on admission, n (%)	23 (25.8)	7 (23.3)	5 (16.7)	11 (37.9)	0.163
SOFA_0_, median (IQR)	1 (0–2)	1 (0–2)	1 (0–1)	2 (1–3)	**0.014**
Apache II_0_, median (IQR)	6 (5–9)	6 (4–7)	7 (5–10)	7 (5–9)	0.058

Significant values are in bold.

*APACHE II* acute physiology and chronic health evaluation II score, *APACHE II*_*0*_ APACHE II score on admission, *BMI* body mass index, *DKA* diabetic ketoacidosis, *DTD* from diagnosis to completion of one DFPP session, *IQR* interquartile range, *OF* organ failure, Obesity, BMI ≥ 28 kg/m^2^, *POF* persistent organ failure, *SOFA* sequential organ failure assessment, *SOFA*_*0*_ SOFA score on admission.

### DFPP treatment and TG clearance

Of the 89 patients, there were no serious complications of DFPP. Twenty-four patients (26.97%) received more than one session of DFPP. The median volume of plasma processed during a DFPP session was 5 L (IQR 3–5 L), equivalent to a median of 2.64 L (IQR 2.34–2.95 L) of the estimated plasma volume (EPV = [0.065 × weight (kg)] × [1-Hct]). The median treatment duration was 200 min (IQR 147.5–250 min), and the median percent decline in TG was 73.63% (IQR 61.12–83.37%). The variables of the DFPP session among the 89 HTG-AP patients sorted by the DTD time tertiles were not significantly notable, as noted in Table 3.

**Table 3:** DFPP treatment and TG clearance during the first 48 h after diagnosis in patients according to DTD time tertiles.

	All patients (n = 89)	T1: ≤ 12 h (n = 30)	T2:13–22 h (n = 30)	T3: ≥ 23 h (n = 29)	P value
DFPP treatment variables					
Frequency of DFPP > 1, n (%)	24 (27)	11 (36.7)	9 (30)	4 (13.8)	0.127
TG clearance rate, median (IQR), %	73.63 (61.12–83.37)	69.14 (43.3–83.3)	75.45 (64.35–83.22)	74.86 (65.12–84.38)	0.276
Volume of plasma processed, median (IQR), L	5 (3–5)	4.3 (3–5)	5 (3–5)	5 (3–5)	0.524
Treatment duration, median (IQR), min	200 (147.5–250)	180 (140–245)	218 (160–280)	200 (164–235)	0.328
Dynamics of TG levels					
TG_0_, median (IQR), mmol/L	58.3 (39.69–80.05)	63.58 (35.84–80.83)	57.41 (44.44–76.75)	54.26 (40.04–81.61)	0.756
TG_0_ > 56.5 mmol, n (%)	46 (51.7)	16 (53.3)	16 (53.3)	14 (48.3)	0.905
TG_24h_, median (IQR), mmol/L	8.99(4.67–19.35)	7.22 (3.04–14.77)	5.84 (3.14–9.78)	17.95 (16.58–27.64)	**< 0.001**
TG_24h_ < 5.65 mmol/L, n (%)	29 (32.6)	15 (50)	13 (43.3)	1 (3.4)	**< 0.001**
TG_48h_, median (IQR), mmol/L	4.44 (3.2–6.03)	3.91 (3.19–5.41)	4.27 (3.08–6.26)	5 (3.64–7.31)	0.178
TG_48h_ < 5.65 mmol/L, n (%)	67 (75.3)	24 (80)	23 (76.7)	20 (69)	0.603
24 h-TG clearance rate, median (IQR), %	83.25 (69.53–91.45)	86.15 (80–91.51)	89.61 (83.01–94.44)	52.54 (42.38–73.66)	**< 0.001**
48 h-TG clearance rate, median (IQR), %	91.81 (87.91–94.02)	91.95 (88.59–94.69)	91.91 (87.96–94.21)	91.4 (86.46–92.81)	0.358

Significant values are in bold.

*DFPP* double filtration plasmapheresis, *DTD* from diagnosis to completion of one DFPP session, *IQR* interquartile range, *POF* persistent organ failure, *TG* triglycerides, *TG*_*0*_ the level of triglycerides at presentation, *TG*_*24h*_ the level of triglycerides at 24 h after diagnosis, *TG*_*48h*_ the level of triglycerides at 48 h after diagnosis, 24 h-TG clearance rate calculated as TG_0_ − TG_24h_/TG_0_; 48 h-TG clearance rate calculated as TG_0_ − TG_48h_/TG_0_.

**Figure 2:**
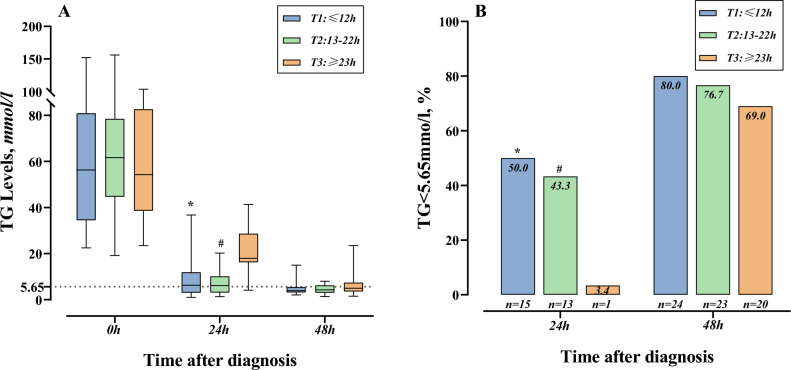
Dynamics of TG levels (**A**) and TG clearance rate (**B**) during the 48 h after diagnosis among the tertiles of DTD time. The Mann‒Whitney U test with a Bonferroni correction was used for multiple comparisons of the TG level and the TG clearance rate: *significant correlation between T1 and T3, #significant correlation between T2 and T3. TG, triglycerides; DTD, from diagnosis to completion of one DFPP session.

The overall reduction in TG levels was from a median of 58.3 mmol/l (IQR 39.69–80.05 mmol/L) at diagnosis to 8.99 mmol/L (IQR 4.67–19.35 mmol/L) at 24 h after diagnosis with a median clearance rate of 83.25% (IQR 69.53–91.45%) and then to 4.4 mmol/L (IQR 3.2–6.03 mmol/L) at 48 h after diagnosis with a median clearance rate of 91.81% (IQR 87.91–94.02%). The number of patients with TGs less than 5.65 mmol/L were 29 (32.6%) at 24 h and 67 (75.3%) at 48 h after diagnosis. As shown in Table 3, the median TG levels at diagnosis were not significantly different among the three groups (P = 0.756). Similarly, the median TG levels, the rate of TG clearance and the proportion of TG < 5.65 mmol/L at 48 h after diagnosis were not significantly different among the three groups (all P > 0.05). However, significant differences were found in the median TG levels, the rate of TG clearance and the proportion of TG < 5.65 mmol/L at 24 h after diagnosis. The median TG levels at 24 h after diagnosis were significantly higher, and TG clearance rates and proportions of TG < 5.65 mmol/L were significantly lower in the highest tertile group than in the other two tertile groups (all P < 0.001; Fig. 2).

### Clinical outcomes

Of the 89 patients, 46 (51.69%) developed POF in the first week of the disease. In addition, 57 and 6 patients (64.0% and 6.7%) developed PNec and IPN, respectively. There were 29 patients who needed ICU support (32.6%), with 18 (20.2%) needing ventilation, 22 (24.7) needing CRRT and 8 (9%) needing vasopressors. There was a total of 2 deaths in this study (mortality rate 5.71%). The 2 deaths occurred in both patients with POF and DTD time ≥ 23 h on day 21 and day 87 of admission, respectively. There were significant differences in the proportions of POF, PNec, and ICU support, including ventilation and CRRT support, among the three groups (all P < 0.05). The proportion of POF and ICU support was significantly higher in the highest tertile group than in the other tertiles. The incidence of PNec was only higher in the highest tertile group than in the lowest tertile group. No significant difference was found in the rate of IPN and mortality (all P > 0.05).

Additionally, the median (IQR) hospital stay was 10 (7–14) days, the hospital charges were 3.5 (2.5–5.2) million yuan, and the APACHE II score and maximum SOFA score at 48 h after admission were 13 (9–15) and 2 (1–3), respectively. The above results differed significantly among the three groups. The patients in the highest tertile group had significantly higher hospital stays, hospital charges and SOFA_max_ scores than those in the other tertile groups (all P < 0.05, Supplementary Fig. S1). The APACHE II scores were only higher in the highest tertile group than in the lowest tertile group (P < 0.05). The main clinical outcomes are shown in Table 4.

**Table 4:** Clinical outcomes of patients according to DTD time tertiles.

	All patients (n = 89)	T1: ≤ 12 h (n = 30)	T2: 13–22 h (n = 30)	T3: ≥ 23 h (n = 29)	P value
POF, n (%)	46 (51.7)	10 (33.3)	12 (40)	24 (82.8)	**< 0.001**
ICU support, n (%)	29 (32.6)	6 (20)	7 (23.3)	16 (55.2)	**0.007**
Ventilation, n (%)	18 (20.2)	2 (6.7)	4 (13.3)	12 (41.4)	**0.002**
CRRT, n (%)	22 (24.7)	4 (13.3)	4 (13.3)	14 (48.3)	**0.002**
Vasopressor, n (%)	8 (9)	1 (3.3)	3 (10)	4 (13.8)	0.347
PNec, n (%)	57 (64.0)	14 (46.7)	18 (60.0)	25 (86.2)	**0.006**
IPN, n (%)	6 (6.7)	1 (3.3)	1 (3.3)	4 (13.8)	0.268
In-hospital mortality, n (%)	2 (2.2)	0 (0)	0 (0)	2 (6.9)	0.107
Hospital stay, median (IQR), day	10 (7–14)	7 (6–11)	9 (7–12)	15 (11–19)	**< 0.001**
Hospital charges, median (IQR), CNY million yuan	3.5 (2.5–5.2)	2.7 (1.9–4.2)	3.0 (2.6–4.2)	5.2 (3.5–8.4)	**< 0.001**
Apache II_48h_, median (IQR)	13 (9–15)	10 (9–13)	13 (10–15)	14 (12–18)	**0.004**
SOFA_48hmax_, median (IQR)	2 (1–3)	1 (1–2)	2 (1–2)	2 (2–4)	**0.003**

Significant values are in bold.

*APACHE II* acute physiology and chronic health evaluation II score, *APACHE II*_*48h*_ APACHE II score during 48 h after admission, *CRRT* continuous renal replacement therapy, *CNY* China Yuan, *DTD* from diagnosis to completion of one DFPP session, *ICU* intensive care unit, *IPN* infected pancreatic necrosis, *IQR* interquartile range, *POF* persistent organ failure, *PNec* pancreatic necrosis, *SOFA* sequential organ failure assessment, *SOFA*_*48hmax*_ maximum SOFA score during 48 h after admission.

### Association between DTD time and POF

The odds ratios (ORs) for POF were lower with decreasing DTD time tertiles (P for trend < 0.001) in the univariable logistic regression analysis (Model 1), and the OR (95% CI) was 0.089 (0.026–0.309) in the lowest DTD time tertile (referencing 1.00 in the highest DTD time tertile). The DTD time-POF association was not materially changed (P for trend < 0.01) in the multivariate logistic regression analysis by further controlling for alcohol abuse, hospital transfer and time from onset to diagnosis (Model 2), as well as additionally adjusting for SOFA and Apache II scores on admission (Model 3) (all P for trend < 0.01). The ORs (95% CI) were 0.042 (0.006–0.285) and 0.061 (0.008–0.452), respectively, in the lowest DTD time tertile (referencing 1.00 in the highest DTD time tertile). The results of ORs for the association of POF with DTD time are shown in Table 5.

**Table 5:** Multivariate logistic regression analyses of the association of DTD time tetiles with the risk of POF.

Model	T1 (n = 30)	T2 (n = 30)	T3 (n = 29)	P value for trend
Model 1—OR (95% CI)	0.089 (0.026–0.309)*	0.159 (0.048–0.531)*	Ref	**< 0.001**
Model 2—OR (95% CI)	0.042 (0.006–0.285)*	0.076 (0.015–0.387)*	Ref	**0.002**
Model 3—OR (95% CI)	0.061 (0.008–0.452)*	0.079 (0.015–0.419)*	Ref	**0.006**

Significant values are in bold.

*P < 0.05.

Model 1 Univariable logistic regression analysis.

Model 2 Adjusted for alcohol abuse, hospital transfer and time from onset to diagnosis.

Model 3 Adjusted for alcohol abuse, hospital transfer, time from onset to diagnosis, SOFA and Apache II scores on admission.

*CI* confidence interval, *DTD* from diagnosis to completion of one DFPP session, *ICU* intensive care unit, *OR* odd ratio, *POF* persistent organ failure.

**Figure 3:**
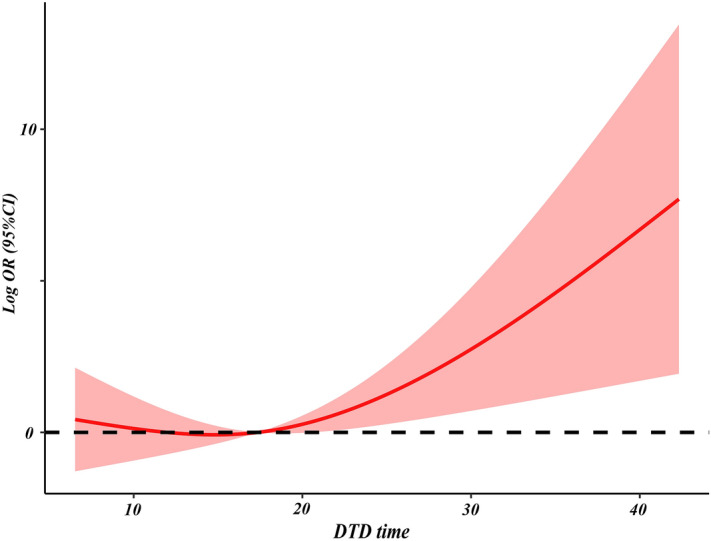
Association of DTD time on a continuous scale and the risk of POF by restricted cubic spline. The red solid line represents the log OR, and the red shaded area represents the 95% confidence interval. The model was adjusted for alcohol abuse, hospital transfer, time from onset to diagnosis, and SOFA and Apache II scores on admission. *CI* confidence interval, *DTD* from diagnosis to completion of one DFPP session, *OR* odds ratio, *POF* persistent organ failure.

Additionally, RCS model analysis was performed by using 3 knots on a total of 89 patients. After adjusting for alcohol abuse, hospital transfer, time from onset to diagnosis, SOFA and Apache II scores on admission, the RCS curves displayed a nonlinear relationship between DTD time and the risk of POF (P for all = 0.0338). The risk of POF was relatively low and flat until approximately 15 h of DTD time and then started to increase rapidly afterwards (P for nonlinearity = 0.0295; Fig. 3).

### Subgroup analyses

**Figure 4:**
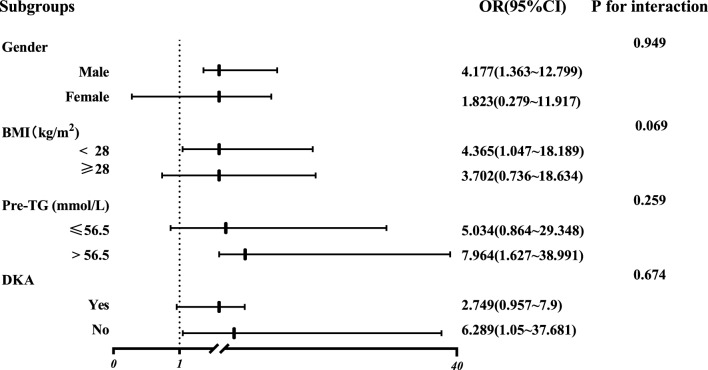
Subgroup analyses of the associations between DTD time and risk of POF. The model was adjusted for alcohol abuse, hospital transfer, time from onset to diagnosis, and SOFA and Apache II scores on admission. Subgroup variables were excluded from the model. *BMI* body mass index, *CI* confidence interval, *DKA* diabetic ketoacidosis, *DTD* from diagnosis to completion of one session of DFPP, *OR* odds ratio, *POF* persistent organ failure, *pre-TG* the level of triglycerides at diagnosis.

When exploring the differences in the subgroups, the participants were stratified by sex (male or female), BMI (< 28 or ≥ 28 kg/m^2^), pre-TG (≤ 56.5 mmol/L or > 56.5 mmol/L), and DKA (no or yes). The subgroup analyses indicated that the ORs for POF were significantly related to DTD time in the male (P = 0.012), BMI ≥ 28 kg/m^2^ (P = 0.043), pre-TG > 56.5 mmol/L (P = 0.01) and DKA groups (P = 0.044) after full adjustment for alcohol abuse, hospital transfer, time from onset to diagnosis, SOFA and Apache II scores on admission, and no significant interaction was observed between DTD time and subgroup variables (Fig. 4).

## Discussions

This study was conducted to understand the relationship between the timing of DFPP initiation and the risk of POF in an HTG-AP cohort in China. To our knowledge, this is the first study to incorporate initiation time into a measure of DFPP treatment and to analyse its impact on outcomes in HTG-AP patients. The principal finding of this study was that early initiation of DFPP treatment was able to reduce the risk of POF in HTG-AP patients. We found an increasing trend for the prevalence of POF with delayed DFPP treatment in the tertiles of DTD time. A similar increasing trend for the incidence of POF was found in the male, BMI ≥ 28 kg/m^2^, pre-TG > 56.5 mmol/L and non-DKA subgroups with increasing tertiles of DTD time. Those associations persisted after adjustment for relevant potential confounding factors. In addition, we used multivariable regression analyses and RCS regression analyses and revealed nonlinear relationships between DTD time and the risk of POF in HTG-AP patients. HTG-AP patients had a relatively low risk of POF when a session of DFPP treatment was completed less than 15 h after diagnosis; since then, the risk of POF has increased rapidly with increasing DTD time.

It has been generally hypothesized that the therapeutic effect of DFPP treatment is based on the immediate physical extracorporeal elimination of TG-rich lipoproteins from plasma, subsequently reducing other pathophysiological processes of pancreatitis, such as inflammation, oxidative stress, and impaired rheology^4,5,12–14^. Thus, the theoretical effect of DFPP was to instantly stop further organ damage and dysfunction in the setting of HTG-AP. Isabel and coworkers found a significant decrease in the APACHE II score after DFPP treatment in four patients with severe HTG-AP, which indicated a decrease in the severity of organ dysfunction^22^. Chang et al. performed nested case‒control studies for HTG-AP patients with or without DFPP. They concluded that DFPP could shorten hospitalization and reduce disease-related complications in HTG-AP patients with serum TG above 56.5 mmol/L (5000 mg/dL)^23^. They also found that prophylactic DFPP reduced the incidence of recurrent HTG-AP, which had been reported in a previous study^23,24^. They thought that the beneficial effect of DFPP therapy was not only due to the rapid reduction in serum triglycerides but also due to the removal of inflammatory lipoproteins (SAA1) and a decrease in oxidative stress^23^. In this study, we showed a promising effect in reducing serum TG levels, which were decreased by 73.6% after a session of DFPP, in accordance with previous studies. We also found that early initiation of DFPP treatment could enable rapid TG reduction to below 5.65 mmol/L within 48 h as soon as possible, and earlier DFPP treatment was associated with lower SOFA_max_ and APACHE II scores at 48 h of admission and less hospital stay and charges. Importantly, after adjustment for potential confounders, the data from our study provided the first clinical evidence that early initiation of DFPP treatment could reduce the risk of POF.

However, the current evidence is too limited to identify the effect of DFPP on beneficial outcomes in HTG-AP patients. DFPP has never been recommended as the preferred treatment for HTG-AP by guidelines around the world because of inconsistent results in several clinical studies. Chen et al. compared two groups of patients before and after apheresis treatment in a large retrospective study and found no benefit on all-cause mortality and complications^28^. They thought the reason was that the time of plasmapheresis might be the critical point. If patients with hyperlipidemic pancreatitis could receive plasma exchange as soon as possible, better result might be expected^28^. If clinical therapy to remove TG from plasma is delayed, the damage from high levels of TG and its derived FFA persists, and a high risk of POF remains^29^. In our study, all the HTG-AP patients with DFPP treatment achieved similar median TG levels and proportions of serum TG less than 5.65 mmol/L within 48 h of diagnosis, but patients with delayed DFPP treatment achieved a high incidence of POF. This means that delayed DFPP treatment loses its benefit of rapid TG clearance because persistent high levels of serum TG and FFA may have already damaged the pancreas and triggered systemic organ damage. The RCS curves showed that the optimal cut-off time for the preventive effect of DFPP treatment on POF was approximately 15 h after diagnosis. This time frame requires further investigation, but it suggests that the timing of DFPP initiation may be critical in reducing the risk of POF in HTG-AP patients.

The initial TG level should be taken into account when assessing the value of early initiation of DFPP in the treatment of HTG-AP. Lu et al. published a propensity score-matched retrospective comparison of HTG-AP patients treated with DFPP and without DFPP. They showed a 74.2% reduction in TG levels after a session of DFPP, similar to our study, but no beneficial effects on clinical outcomes between the groups^26^. The reason for the negative conclusion was considered to be due to the difference in the initial TG levels^30^. In Lu’s study, the mean TG levels in patients without DFPP treatment were reduced from 31.1 mmol/L (2755 mg/dL) on admission to 5.4 mmol/L (478 mg/dL) at 48 h, with no difference from those treated with DFPP. This meant that DFPP was not necessary because conservative management alone was able to reduce the TG levels sufficiently with such initial TG levels^26,30^. In the subgroup of our study, we found that early DFPP treatment could reduce the risk of POF only in patients with pre-TG levels above 56.5 mmol/L (5000 mg/dL). This is because conservative management may become insufficient to reduce TG in a timely manner as initial TG levels become higher. In a study of conservative management in HTG-AP patients, the average TG level was reduced from 45.4 mmol/L on presentation to 13.3 mmol/L within 48 h, corresponding to a 48-h reduction of 69.8%, which was similar to a session of DFPP, but could still not keep the 48-h TG level away from the threshold for the risk of POF^31^. In this condition, it is reasonable to initiate early DFPP treatment for rapid TG lowering. It is necessary to focus on the initial TG level, which should be the indication for early initiation of DFPP in the treatment of HTG-AP.

In addition, the effect of early DFPP treatment on reducing the risk of POF may be influenced by the other initial conditions, which have been reported to be associated with the severity of AP. Males were found to have significantly worse clinical outcomes in AP^32^, and BMI ≥ 28 kg/m^2^ might amplify the inflammatory response of pancreatic and peripancreatic tissue and increase the risk of organ failure^33,34^. In our study, early DFPP treatment showed a significant trend to reduce the risk of POF in the male and obese subgroups but not in the female and nonobese subgroups. This suggests that early initiation of DFPP may be more meaningful for male patients or those with BMI ≥ 28 kg/m^2^. Interestingly, it was reported that DM should aggravate the severity of AP^35,36^, but in our study, the early completion of DFPP treatment was not significantly associated with a reduced risk of POF in the DKA subgroup with HTG-AP. As we used fluid infusion and insulin therapy to treat DKA in HTG-AP patients, which was also an effective TG-lowering treatment, the benefit of DFPP treatment might be attenuated. In conclusion, DFPP is one of the most important TG-lowering treatments in clinical practice. Early initiation of DFPP treatment is beneficial and preferred according to individual conditions such as pre-TG level, sex, BMI ≥ 28 kg/m^2^ or other initial comorbidities.

However, our study also had several inherent limitations. First, we had no corresponding non-DFPP treatment group to provide direct comparison, so the study by default was restricted, and the results might be biased. Second, we chose the time from diagnosis to DFPP rather than the time from onset to DFPP because of the objectivity of the diagnosis time. The conclusion might be influenced by the bias of different individuals from onset to diagnosis time, although the role of this factor was attenuated by multivariate logistic regression. Third, our analyses of patients with HTGP were limited by relatively small sample sizes, which underpowered our ability to assess outcomes, especially the rare outcomes of in-hospital mortality and IPN. In addition, the patients enrolled in the study were from a large university-affiliated tertiary care hospital and could not represent the entire population of patients with HTG-AP; thus, the results cannot be generalized. Fourth, due to the retrospective nature of the study, it is subject to all the biases associated with retrospective studies. Therefore, the effect of DFPP on HTG-AP outcomes should be examined further in well-designed prospective studies or randomized clinical trials. Moreover, DFPP therapy was decided by the treating clinicians. Different DFPP treatment dosages were not discussed in this study, which needs further exploration.

## Conclusions

In this retrospective cohort study, we confirmed that the early initiation of DFPP treatment correlated with a reduced risk of POF in HTG-AP patients. Early initiation of DFPP treatment could also show some beneficial effects on decreasing disease severity characterized by the incidence of PNec, the need for ICU support, SOFA_max_ and APACHE II scores at 48 h of admission, as well as hospital stay and costs. Based on our study, the optimal time to initiate DFPP treatment was approximately 15 h after confirmed diagnosis; otherwise, the beneficial effect of DFPP treatment in reducing the risk of POF might be significantly attenuated. In addition, our findings suggested that HTG-AP patients with male sex, BMI ≥ 28 kg/m^2^, non-DKA, or initial TG levels above 56.5 mmol/L (5000 mg/dL) should be preferred for early initiation of DFPP treatment. However, well-designed randomized controlled trials are needed to confirm these results.

### Supplementary Information

Supplementary Figure S1.

## Data Availability

All data generated and analyzed in this study are included in this published article. The datasets are available from the corresponding author on reasonable request.

## Abbreviations

AP: Acute pancreatitis
APACHE II: Acute Physiology and Chronic Health Evaluation score II
BMI: Body mass index
CI: Confidence interval
CRRT: Continuous renal replacement therapy
CNY: China Yuan
DFPP: Double filtration plasmapheresis
DKA: Diabetic ketoacidosis
DTD: From diagnosis to completion of one DFPP session
FFAs: Free fatty acids
FFP: Fresh frozen plasma
ICU: Intensive care unit
IPN: Infected pancreatic necrosis
IQR: Interquartile range
HTG: Hypertriglyceridemia
HTG-AP: Hypertriglyceridemia-induced acute pancreatitis
OF: Organ failure
OR: Odd ratio
POF: Persistent organ failure
PNec: Pancreatic necrosis
RCS: Restricted cubic spline
SAP: Severe acute pancreatitis
SOFA: Sequential organ failure assessment score
TG: Triglycerides
TPE: Therapeutic plasmapheresis
