# Supplementary material for: Initiation time of double-filtration plasma apheresis affects the risk of persistent organ failure in hypertriglyceridaemia-induced acute pancreatitis: a retrospective study
Source: Sci Rep. 2023 Aug 10;13:13003. doi: 10.1038/s41598-023-40287-2 (PMC10415268; doi:10.1038/s41598-023-40287-2)
Supplement: Supplementary file 1 — Supplementary Figure S1. [file 41598_2023_40287_MOESM1_ESM.pdf]

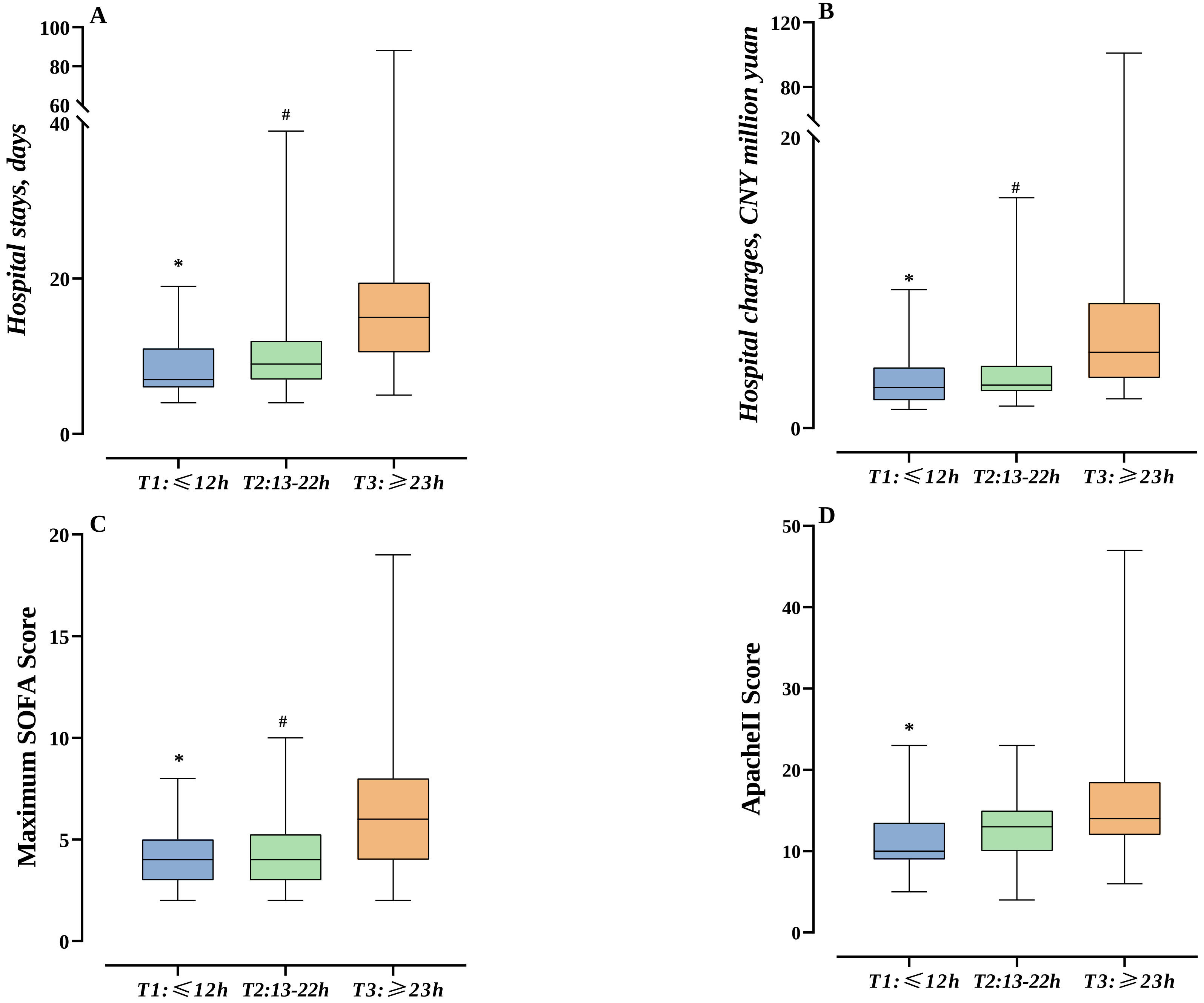

Fig. S1 Hospital stay（A）、Hospital changes（B）、Maximum SOFA Score（C）and ApacheII Score（D）by 48h after admission among the tertiles of DTD time

Mann-Whitney U test with a Bonferroni correction was used for multiple comparisons: * significant correlation between T1 and T3, # significant correlation between T2 and T3. APACHE II, acute physiology and chronic health evaluation II score; CNY, China Yuan; DTD, from diagnosis to completion of one DFPP session; SOFA, sequential organ failure assessment score.
